# Exosome-shuttled miR-150–5p from LPS-preconditioned mesenchymal stem cells down-regulate PI3K/Akt/mTOR pathway via Irs1 to enhance M2 macrophage polarization and confer protection against sepsis

**DOI:** 10.3389/fimmu.2024.1397722

**Published:** 2024-06-18

**Authors:** Ting Zheng, Sipeng Li, Teng Zhang, Wei Fu, Shuchang Liu, Yuxin He, Xiao Wang, Tao Ma

**Affiliations:** ^1^ Department of General Surgery, Tianjin Medical University General Hospital, Tianjin, China; ^2^ Department of Orthopedics, The First Affiliated Hospital of Shandong First Medical University and Shandong Provincial Qianfoshan Hospital, Jinan, China

**Keywords:** sepsis, exosomes, macrophage, miR-150-5p, Irs1

## Abstract

**Rationale:**

Sepsis is a life-threatening organ dysfunction and lack of effective measures in the current. Exosomes from mesenchymal stem cells (MSCs) reported to alleviate inflammation during sepsis, and the preconditioning of MSCs could enhance their paracrine potential. Therefore, this study investigated whether exosomes secreted by lipopolysaccharide (LPS)-pretreated MSCs exert superior antiseptic effects, and explored the underlying molecular mechanisms.

**Methods:**

Exosomes were isolated and characterized from the supernatants of MSCs. The therapeutic efficacy of normal exosomes (Exo) and LPS-pretreated exosomes (LPS-Exo) were evaluated in terms of survival rates, inflammatory response, and organ damage in an LPS-induced sepsis model. Macrophages were stimulated with LPS and treated with Exo or LPS-Exo to confirm the results of the *in vivo* studies, and to explain the potential mechanisms.

**Results:**

LPS-Exo were shown to inhibit aberrant pro-inflammatory cytokines, prevent organ damages, and improve survival rates of the septic mice to a greater extent than Exo. *In vitro*, LPS-Exo significantly promoted the M2 polarization of macrophages exposed to inflammation. miRNA sequencing and qRT-PCR analysis identified the remarkable expression of miR-150–5p in LPS-Exo compared to that in Exo, and exosomal miR-150–5p was transferred into recipient macrophages and mediated macrophage polarization. Further investigation demonstrated that miR-150–5p targets Irs1 in recipient macrophages and subsequently modulates macrophage plasticity by down-regulating the PI3K/Akt/mTOR pathway.

**Conclusion:**

The current findings highly suggest that exosomes derived from LPS pre-conditioned MSCs represent a promising cell-free therapeutic method and highlight miR-150–5p as a novel molecular target for regulating immune hyperactivation during sepsis.

## 1 Introduction

Sepsis is defined as a life-threatening organ dysfunction caused by dysregulated host immune and inflammatory responses (1). It is a common and major cause of morbidity and mortality in intensive care units. Despite advances in critical care, the global incidence of sepsis is estimated at 18 million cases per year, with a mortality rate of between 30% and 50% for severe sepsis (2, 3). To date, no agents have been reported to be specifically approved to treat sepsis. Thus, effective treatment regimens remain elusive. Macrophages play a crucial role in regulating the host’s immune balance and inflammatory response in sepsis. In response to prevailing stimuli within inflammatory microenvironments, macrophages can become polarized toward either pro-inflammatory M1 or anti-inflammatory M2 phenotype, respectively. M1 macrophages exhibit a robust inflammatory response and are capable of killing pathogens, while M2 macrophages promote tissue repair and resolution of inflammation (4, 5). In sepsis, there is excessive activation of M1 macrophages and inadequate activation of M2 macrophages, leading to persistent inflammatory response and tissue damage (6, 7). Therefore, investigating the regulation of macrophage polarization, especially new therapeutic strategies that promote M2 macrophage polarization, is of research value for the treatment of sepsis.

Mesenchymal stem cells (MSCs) have been shown to possess immunomodulatory and tissue regeneration capacities, and have emerged as a promising therapeutic approach in many inflammatory disease (8, 9). However, the safety and immunological rejection of MSCs transplantation limits its clinical application (10, 11). Currently, increasing data indicated that MSCs create an optimal microenvironment for reducing inflammation through a paracrine mechanism and that exosomes are crucial in this process (12), indicating that transfusion of exosomes may be an alternative option for MSC-based therapy (13, 14).

Exosomes are a kind of extracellular vesicles with a diameter of 30 to 150 nm, which can delivery bioactive molecules to recipient cells and modulate cellular activities and the phenotype of recipient cells (15). Exosomes have been confirmed as the main player accounting for the paracrine effects of MSCs. Similar functions to their parental cells have been observed in MSC-exosomes, such as antimicrobial effects, immunosuppression, and regeneration ability (16, 17). Compared with MSCs, exosomes possess hypoimmunogenic properties, low tumorigenicity, and higher stability (18, 19). Studies have shown that exosomes play a therapeutic role in various inflammatory diseases, primarily by delivering protein or miRNAs (20, 21). Specifically, exosomal miR-30d-5p from Polymorphonuclear neutrophils (PMNs) contributed to sepsis-related acute lung injury (ALI) by inducing M1 macrophage polarization and priming macrophage pyroptosis via NF-κB activation (22). Moreover, LPS preconditioning improves the regulatory abilities of MSC-derived exosomes for macrophage polarization by shuttling let-7b, which hold significant immunotherapeutic potential for wound healing (21).

Recent studies have proven that exosomes from MSCs can improve the outcome of sepsis, and appropriate preconditioning can enhance the paracrine ability of MSCs to improve their therapeutic potential (23–25). Here, we provide a new strategy to effectively enhance the therapeutic effect of MSC-derived exosomes against sepsis by preconditioning MSCs with lipopolysaccharide (LPS) and elucidate the molecular mechanism.

## 2 Materials and methods

### 2.1 Culture and treatment of MSCs

Adipose-derived MSCs (ADSCs) were obtained from C57BL/6J mice following the procedure previously reported (26). The isolated cells were resuspended in Dulbecco’s modified eagle medium (DMEM)/F12 (Gibco) containing 10% exosome-depleted fetal bovine serum (FBS, Gibco) (160,000 ×g at 4 °C, overnight) (27), and 100 U/mL penicillin/streptomycin (Gibco). The immunophenotype of MSCs were detected using flow cytometry. And their multilineage differentiation was confirmed by osteogenic and adipogenic differentiation. Cells at 3^rd^ to 5^th^ passages were used for further experiments. Preconditioning with LPS (Sigma) was performed once the cells reached 70–80% confluence. MSCs were cultured in fresh medium supplemented with LPS (1 μg/mL) or PBS for 48 h before supernatant collection.

### 2.2 Isolation and characterization of exosome

Exosomes were isolated from the supernatants of MSCs and LPS-treated MSCs using differential ultracentrifugation as previously described (28). Cell supernatants were centrifuged at 350 ×g for 10 min to remove dead cells, 2,000 ×g for 10 min to remove cell debris, and 10,000 ×g for 30 min at 4°C to remove microvesicles. Next, the supernatant was filtered through a 0.22 μm filter and centrifuged at 120,000 ×g for 70 min at 4 °C. The pallets were re-suspended in PBS and centrifuged at 120,000 ×g for 70 min at 4 °C again. The final pellets were re-suspended with PBS and stored at -80 °C for further experiment.

The morphology of the isolated exosomes was observed with transmission electron microscopy (TEM) (Hitachi, HT7700, Japan). The particle size distribution and concentration of exosomes were analyzed with nanoparticle tracking analysis (NTA) (Malvern, NS300, UK). The specific markers CD9, CD63, and TSG101 were detected using western blot. The protein concentration was quantified using a BCA protein assay (Applygen).

### 2.3 Sepsis model and exosome therapy

C57BL/6J male mice, 6–8 weeks old, were purchased from Vital River Laboratory Animal Technology (Beijing, China). All animal experiments carried out in accordance with the National Institutes of Health guide for the care and use of Laboratory animals, and approved by the Animal Care and Use Committee of the General Hospital, Tianjin Medical University.

The mice were randomly divided into groups and then intraperitoneally injected with LPS (12.5 mg/kg), and the mice in the control group were given the same dose of PBS. Two hours after the LPS injection, mice were treated with LPS-pretreated exosomes (LPS-Exo, 200 μg/mouse), normal exosomes (Exo, 200 μg/mouse), or PBS through the tail vein injection. The same dose of PBS was administered to the mice in the control group. Status of survival of the mice were monitored every 6 h. Serum samples were collected 12 h after LPS injection. Mice were euthanized 48 h after LPS injection, and organ damage was examined. Mice were anesthetized with isopentane and sacrificed by cervical dislocation.

### 2.4 BMDMs culture and treatment

BMDMs were obtained from C57BL/6J mice as previously described (29). The isolated cells were cultured in DMEM with 10% FBS, 100 U/ml penicillin/streptomycin, and 10 ng/mL M-CSF (Pepro Tech) in 5% CO_2_ at 37 °C for 7 days. The BMDMs were stimulated with LPS (1 μg/mL) to establish an *in-vitro* sepsis model and then treated with Exo, LPS-Exo, or PBS.

To trace the internalization of exosomes by BMDMs, exosomes were labeled with DiI (MedChemExpress), washed with PBS, ultracentrifuged and resuspended in PBS. After being incubated with DiI-labeled exosomes for 12 h, the BMDMs were rinsed with PBS, fixed for 30 min in 4% paraformaldehyde, and stained with DAPI. Finally, the cellular uptake of exosomes was detected using a laser-scanning confocal microscope (Zeiss LSM 800, Germany).

### 2.5 RNase and proteinase K treatment of LPS-Exo

To investigate the molecular cargo of LPS-Exo which improved sepsis. LPS-Exo was treated with proteinase K (100 μM/mL, Sigma) for 30 min at 37 °C or with RNase A (100 μM/mL, Sigma) for 15 min at 37 °C. Exosome-free proteins or RNA was used for further experiments.

### 2.6 Exosomal miRNA sequencing and bioinformatics analysis

Total RNA of the exosomes was extracted using the miRNeasy Serum/Plasma Advanced Kit (Qiagen). The final ligation PCR products were sequenced using the Illumina NovaSeq 6000 platform (Echo Biotech, Beijing, China). miRNAs in Exo and LPS-Exo were profiled in three biological replicates. The expression differences between Exo and LPS-Exo were analyzed with a *t*-test. Those with a fold change > 1.5 and a *p*-value < 0.05 were regarded as significantly different expressed.

miRanda and RNAhybrid were used to predict the target genes of the differentially expressed miRNAs. GO categories of the predict target genes were performed using the DAVID bioinformatics database. Pathway analysis was performed using the KEGG database and the 20 most enriched signaling pathways were listed to identify the most relevant signaling pathways.

### 2.7 qRT-PCR

Total RNA of the exosomes was extracted using the miRNeasy Serum/Plasma Advanced Kit. Total RNA of the cells was extracted using the TRIzol reagent. The isolated RNA was reverse transcribed using a cDNA synthesis kit (Qiagen). PCR was performed in a CFX Connect (BIO-RAD) system using SYBR Green PCR Master Mix (Qiagen), using GAPDH or U6 as an internal control. The primer and probe sequences are listed in **Table 1**.

**Table 1:** Primer sequences.

Name of Primer	F Sequences (5’-3’)	R Sequences (5’-3’)
miR-150–5p	TCT CCC AAC CCT TGTA	GAA TAC CTC GGA CCC TGC -
U6	AAA GCA AAT CAT CGG ACG ACC	GTA CAA CAC ATT GTT TCC TCGGA
Arg1	CATTGGCTTGCGAGACGTAGAC	GCTGAAGGTCTCTTCCATCACC
iNOS	GAGACAGGGAAGTCTGAAGCAC	CCAGCAGTAGTTGCTCCTCTTC
GAPDH	CCG CAT CTT CTT GTG CAG TG	ACC AGC TTC CCA TTC TCA GC

F forward; R reverse.

### 2.8 miRNA transfection

miR-150–5p inhibitor and negative control (NC) were purchased from GenePharma and diluted with DEPC water. For miR-150–5p inhibition, BMDMs were transfected with miR-150–5p inhibitor or NC at a concentration of 50 nM using Lipofectamine3000 (ThermoFisher) before treated with Exo or LPS-Exo. MSCs were transfected with miR-150–5p inhibitor or NC at a concentration of 50 nM using Lipofectamine3000 for 6 h and then cultured in fresh medium with LPS (1μm/ml) for further 48 h (30). The exosomes were extracted after transfection as previously described. The transfection efficiency was evaluated by qRT-PCR.

### 2.9 ELISA and NO assay

The concentration of TNF-a, IL-6, and IL-10 in serum and the supernatant of cells was determined using commercial ELISA kits (Biolegend). The levels of NO in the supernatant of the cells were determined using NO kits (Beyotime).

### 2.10 Histological examination

Lung, liver, and kidney tissues were obtained and fixed with 10% paraformaldehyde, embedded in paraffin, and then cut into 5-μm sections. After that, the sections were stained with hematoxylin and eosin for evaluation. Injury areas were analyzed as previously described (31).

### 2.11 Western blotting

Cells and exosomes were isolated and lysed with ice-cold radioimmunoprecipitation assay lysis buffer containing protease and phosphatase inhibitors (Applygen). Protein concentration was tested by a BCA assay. Equal amounts of proteins from each sample were used for immunoblot analysis as previously described (32). Data were analyzed using the ImageJ software. The primary antibodies employed for western blotting are listed in **Table 2**.

**Table 2:** Antibodies.

Name of antibody	Company	Ratio of dilution	Catalogue number
CD9	Abcam	1:1000	ab307085
CD63	Abcam	1:1000	ab217345
TSG101	Abcam	1:1000	ab125011
iNOS	Affinity	1:1000	AF0199
CD206	Cell Signaling Technology	1:1000	24595T
Irs1	Abcam	1:1000	ab131487
Akt	Abcam	1:1000	ab179463
p-Akt	Abcam	1:1000	ab192623
PI3K	Abcam	1:1000	ab191606
p-PI3K	Abcam	1:1000	ab278545
mTOR	Abcam	1:1000	ab134903
p-mTOR	Abcam	1:1000	ab109268
GAPDH	Applygen	1:1000	C1312

### 2.12 Luciferase assay

HEK293T cells (1×10^5^ cells/well) were seeded into a 12-well plate 24 h before transfection. Then, 1 μg pmir-GLO vectors containing a wild-type (WT) or mutant (MUT) fragment of the IRS1 3’UTR, 50 nM miR-150–5p mimic or NC were co-transfected using Lipofectamine3000. Luciferase activity was detected using a Dual-Glo luciferase assay system 24 h after transfection.

### 2.13 Statistical analysis

Data were analyzed using GraphPad Prism 9.0 and presented as mean ± standard deviation. A *t*-test was used to compare the significance between two groups. Survival rates between the groups were compared using the log-rank test. A *p-*value < 0.05 was considered to be significant.

## 3 Results

### 3.1 Extraction and identification of MSCs and exosomes

We first obtained MSCs from the subcutaneous adipose tissue of C57BL/6 mice. The MSCs were exhibited a spindle-shaped morphology under the light microscope (**Supplementary Figure S1A**). Additionally, these cells were capable of differentiating into osteoblasts and adipocytes (**Supplementary Figures S1B, C**). FACS analysis demonstrated that these cells were positive for CD29 and CD44, and negative for CD45 (**Supplementary Figure S1D**). These data indicated that we successfully obtained ADSCs.

**Figure 1:**
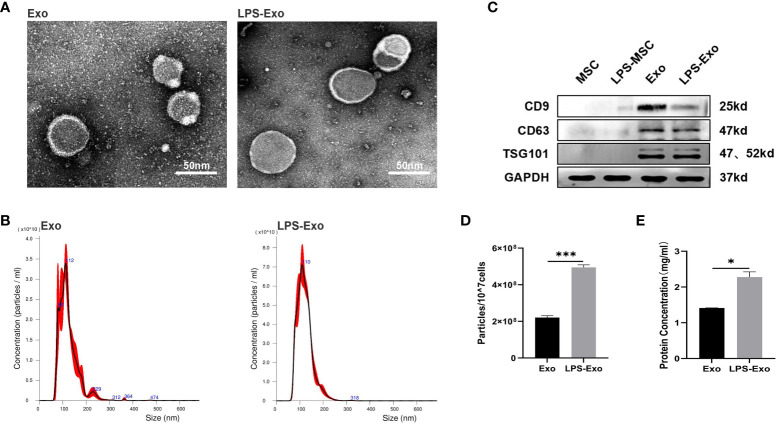
Characterization of Exo and LPS-Exo. **(A)** Representative TEM images of Exo and LPS-Exo. Scale bars: 50 nm. **(B)** Particle size distribution of Exo and LPS-Exo determined by NTA. **(C)** Specific markers (CD9, CD63, and TSG101) were evaluated by western blotting. The enhanced secretion of exosomes was detected by NTA **(D)** and BCA assay **(E)** after LPS pretreatment. Data are presented as the mean ± standard deviation. ^*^ *p* < 0.05, ^***^ *p* < 0.001.

We then collected the supernatants of the LPS-pretreated or non-pretreated MSCs and extracted the exosomes using differential ultracentrifugation. TEM, NTA, and western blotting were performed to verify the characteristics of exosomes. The TEM images showed that LPS-Exo and Exo possessed a typical cup-like appearance with double-membrane structures (**Figure 1A**). NTA results showed that the diameters of Exo and LPS-Exo were between 50 and 200 nm (**Figure 1B**). Western blotting indicated that the specific exosomal markers CD9, CD63, and TSG101 were expressed in Exo and LPS-Exo (**Figure 1C**). These results revealed that LPS-Exo and Exo share identical exosomal characteristics, including shape, size, and biomarkers. However, using BCA and NTA assays, we found significantly higher levels of proteins and particles in LPS-Exo than in Exo, confirming that LPS preconditioning promoted the secretion of exosomes from MSCs (**Figures 1D, E**).

### 3.2 LPS-Exo alleviates sepsis with higher efficacy

**Figure 2:**
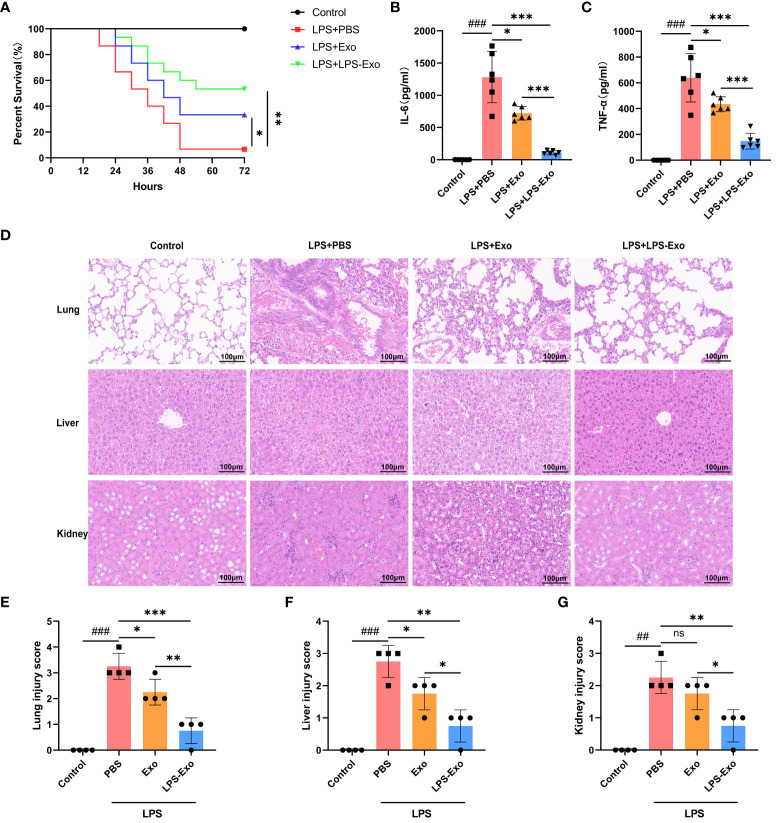
LPS-Exo alleviates sepsis with higher efficacy. **(A)** Survival rate changes of mice in different groups (n=15). Expression levels of IL-6 **(B)** and TNF-α **(C)** in serum (n=6). **(D)** Representative H&E-stained sections of lung, liver, and kidney tissues from mice are shown at ×400 original magnification. Scale bar 100 μm. **(E-G)** Pathological lung, liver, and kidney injury scores of representative samples from mice in different groups (n=4). Data are presented as the mean ± standard deviation. ^*^ *p* < 0.05, ^**^ *p* < 0.01, ^***^ *p* < 0.001, compared to the PBS group. ^##^ *p* < 0.01, ^###^ *p* < 0.001, compared to the control group. ns: p > 0.05.

We injected septic mice via the tail vein with LPS-Exo (200 μg/mouse), Exo (200 μg/mouse), or PBS, and the effect on survival rate was evaluated. Septic mice treated with LPS-Exo and Exo exhibited significantly increased survival rates compared to mice treated with PBS (53.3% vs. 6.7% and 33.3% vs. 6.7%, respectively; *p* < 0.05), and the survival rate in the LPS-Exo group was the highest (**Figure 2A**). As expected, levels of IL-6 and TNF-α in the plasma were increased in the setting of sepsis. Both LPS-Exo and Exo inhibited the increase of these pro-inflammatory cytokines. After exosome treatment, IL-6 and TNF-α levels decreased by 91.4% and 76.8%, respectively, in the LPS-Exo group as well as by 43.9% and 32.1%, respectively, in the Exo group. The reduction of cytokines levels in the LPS-Exo group was significantly greater than that in the Exo group (**Figures 2B, C**).

Multiple organ dysfunction syndrome (MODS) is the most common serious complication of sepsis, associating with high mortality rates. The effects of LPS-Exo and Exo on sepsis-induced MODS were evaluated using histological examination. Indeed, the septic mice exhibited interalveolar septum thickening and alveolar inflammatory cell infiltration in the lungs, diffuse hemorrhage and inflammatory cell infiltration in the kidneys, and multiple hepatic cell vacuolations and steatosis in the liver, as opposed to the control mice (**Figure 2D**). Administration of LPS-Exo or Exo considerably attenuated these organ injury scores, whereas the efficacy of LPS-Exo was better than that of Exo (**Figures 2E-G**). These results indicated that LPS-Exo possessed potent anti-inflammatory activity and protected against sepsis with higher efficacy.

### 3.3 LPS-Exo strongly induces M2 polarization of macrophages *in vitro*

**Figure 3:**
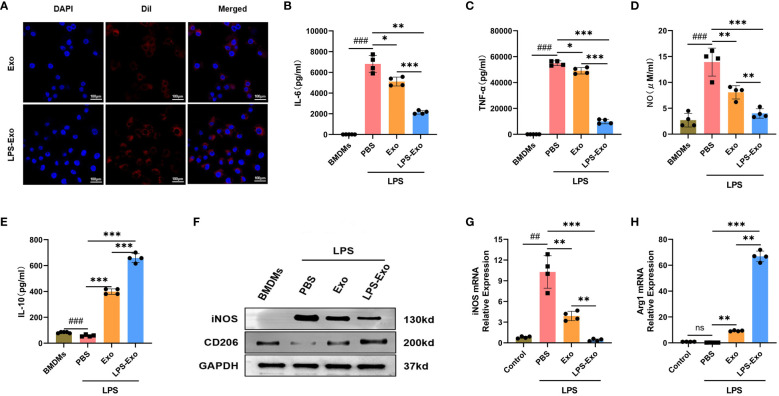
LPS-Exo strongly induces M2 polarization of macrophages *in vitro*. **(A)** Cellular uptake of DiI-labeled exosomes (red) by BMDMs (DAPI blue). Scale bar 100 μm. **(B-E)** Expression levels of IL-6, TNF-α, NO, and IL-10 in the supernatant of LPS-stimulated BMDMs after culturing with LPS-Exo, Exo, or PBS for 12h (n=4). **(F)** Protein expression of iNOS and CD206 in different treatment groups was analyzed by western blot. Gene expression of iNOS **(G)** and Arg1 **(H)** in different treatment groups was analyzed by qRT-PCR (n=4). All data are presented as mean ± standard deviation. ^*^ *p* < 0.05, ^**^ *p* < 0.01, ^***^ *p* < 0.001, compared to the PBS group. ^##^ *p* < 0.01, ^###^ *p* < 0.001, compared to the control group. ns: p > 0.05.

To explore the immunosuppressive mechanism of LPS-Exo, we established an *in vitro* model of sepsis using BMDMs. First, we demonstrated the uptake of LPS-Exo and Exo by BMDMs using confocal microscopy (**Figure 3A**). Then, BMDMs were stimulated with LPS and co-cultured with LPS-Exo, Exo, or PBS for 24 h. The expression levels of inflammatory cytokines in the supernatant of BMDMs were evaluated at 12 h using ELISA. The results indicated that the levels of pro-inflammatory cytokines (IL-6, TNF-α, and NO) were significantly decreased and the anti-inflammatory cytokine (IL-10) was remarkably increased in the LPS-Exo and Exo groups compared with those in the PBS groups, whereas the LPS-Exo had a stronger effect than Exo (**Figures 3B-E**). Western blotting and qRT-PCR were performed to detected the phenotype markers expression of BMDMs at 24 h. The results showed that after treatment with LPS-Exo and Exo, M1 phenotype marker (iNOS) expression was decreased, and the M2 phenotype marker (CD206 and Arg1) expression were increased. And LPS-Exo had a stronger effect than Exo (**Figures 3F-H**). These data indicated that LPS-Exo and Exo treatment promoted M2 polarization and inhibited M1 polarization and that LPS-Exo exhibited a stronger effect than Exo.

### 3.4 Expression pattern of miRNA in LPS-Exo

**Figure 4:**
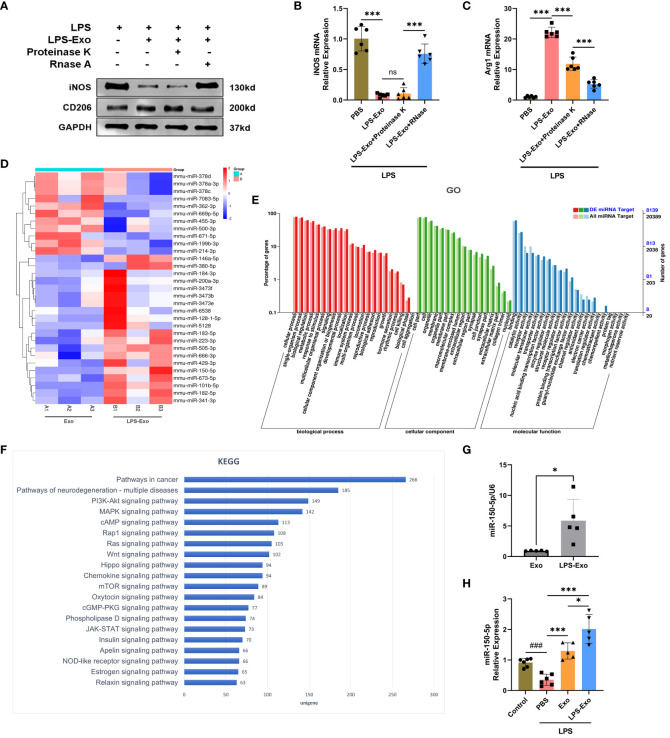
Expression pattern of miRNA in LPS-Exo. **(A-C)** Efficacy of LPS-Exo under proteinase K and RNase treatment on BMDM polarization under LPS stimulation (n=6). **(D)** The heatmap displaying the differentially expressed miRNAs between Exo and LPS-Exo. **(E)** GO categories analysis on differentially expressed miRNAs. **(F)** KEGG analysis was performed based on the predicted target genes of the differentially expressed miRNAs in LPS-Exo. The 20 most enriched pathways associated with signaling transduction are shown. **(G-H)** The qRT-PCR analysis demonstrated the differential expression level of miR-150–5p in Exo, LPS-Exo, and recipient macrophages treated with Exo, LPS-Exo, or PBS (n=5). Data are expressed as the mean ± standard deviation. ^*^ *p* < 0.05, ^***^ *p* < 0.001, compared to the PBS group. ^###^ *p* < 0.001, compared to the control group. ns: p > 0.05.

Given that LPS-Exo possessed a better efficacy than Exo in regulating macrophage polarization, which was primarily through delivering miRNA or protein to recipient cells, we proposed that LPS stimulation changes the composition of LPS-Exo cargo through which it exerts its activity. Thereby, LPS-Exo was processed with proteinase K or RNase to determine the molecular cargo responsible for its immunomodulatory activity. Western blot analysis of iNOS and CD206 showed that LPS-Exo was unaffected by proteinase K treatment, whereas LPS-Exo failed to exert protective effects following RNase treatment (**Figure 4A**). Similar results were obtained using qRT-PCR (**Figures 4B, C**). These results demonstrated that miRNA molecules in LPS-Exo responsibility for its superior immunomodulatory properties.

The miRNA expression profiles of LPS-Exo and Exo were sequenced and compared, and 31 significant differentially expressed miRNAs were found (fold change > 1.5, adjusted *p*-value < 0.05), shown in a hierarchical clustering plot (**Figure 4D**). GO analysis revealed the influence of these differentially expressed miRNAs on various biological functions (**Figure 4E**). KEGG analysis revealed the most enriched signaling pathways relate to these differentiated miRNAs, and our data indicated that the PI3K/Akt and mTOR signaling pathways were within the 20 most enriched pathways (**Figure 4F**).

### 3.5 LPS-Exo delivers miR-150–5p alleviates sepsis

Among the markedly up-regulated differentially expressed miRNAs, miR-150–5p ranked second, which was previously reported to regulate macrophages polarization. We then rectified that the expression of miR-150–5p in LPS-Exo was significantly higher than that in Exo using qRT-PCR (**Figure 4G**). Additionally, macrophages treated with LPS-Exo exhibited higher expression of miR-150–5p (**Figure 4H**).

**Figure 5:**
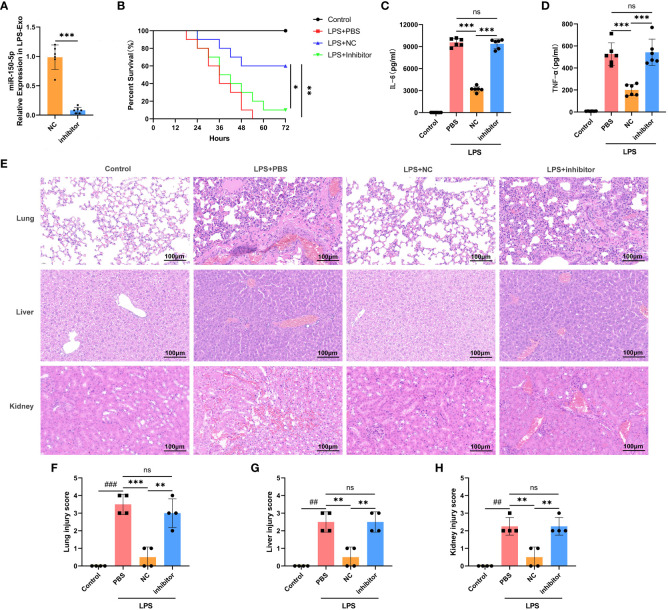
LPS-Exo delivers miR-150–5p alleviates sepsis. **(A)** qRT-PCR analysis demonstrated the transfection efficacy of miR-150–5p inhibitor in LPS-Exo (n=6). **(B)** Survival rate changes of mice in different groups (n=10). Expression levels of IL-6 **(B)** and TNF-α **(C)** in serum were measured at 12h after LPS injection (n=6). **(E)** Representative H&E-stained sections of lung, liver, and kidney tissues from mice in different treatment groups are shown at ×400 original magnification. Scale bar 100 μm. **(F-H)** Pathological lung, liver, and kidney injury scores of representative samples from mice in different groups (n=4). Data are presented as the mean ± standard deviation. ^**^ *p* < 0.01, ^***^ *p* < 0.001, compared to the PBS group. ^##^ *p* < 0.01, ^###^ *p* < 0.001, compared to the control group. ns: p > 0.05.

To investigate the functional role of miR-150–5p in LPS-Exo *in vivo*, we transfected MSCs with miR-150–5p inhibitor or NC prior to LPS stimulation. The exosomes were extracted and named inhibitor LPS-Exo or NC LPS-Exo, and the transfection efficacy was validated by qRT-PCR (**Figure 5A**). We then established an LPS-induced sepsis model and treated via tail vein injection with inhibitor LPS-Exo, NC LPS-Exo, or PBS. The survival rate of septic mice treated with the inhibitor LPS-Exo was significantly lower than that of mice treated with the NC LPS-Exo (**Figure 5B**). ELISA also showed that the protective effect of LPS-Exo on the release of proinflammatory cytokines was blocked by miR-150–5p inhibition (**Figures 5C, D**). Notably, pathological examination showed that downregulation of miR-150–5p in LPS-Exo led to a notable aggravation of the histological damage of organs (**Figures 5E-H**). Collectively, these data demonstrated that LPS-Exo delivers miR-150–5p alleviates sepsis.

### 3.6 Exosomal miR-150–5p modulates the PI3K/Akt/mTOR signaling pathway via targeting Irs1

**Figure 6:**
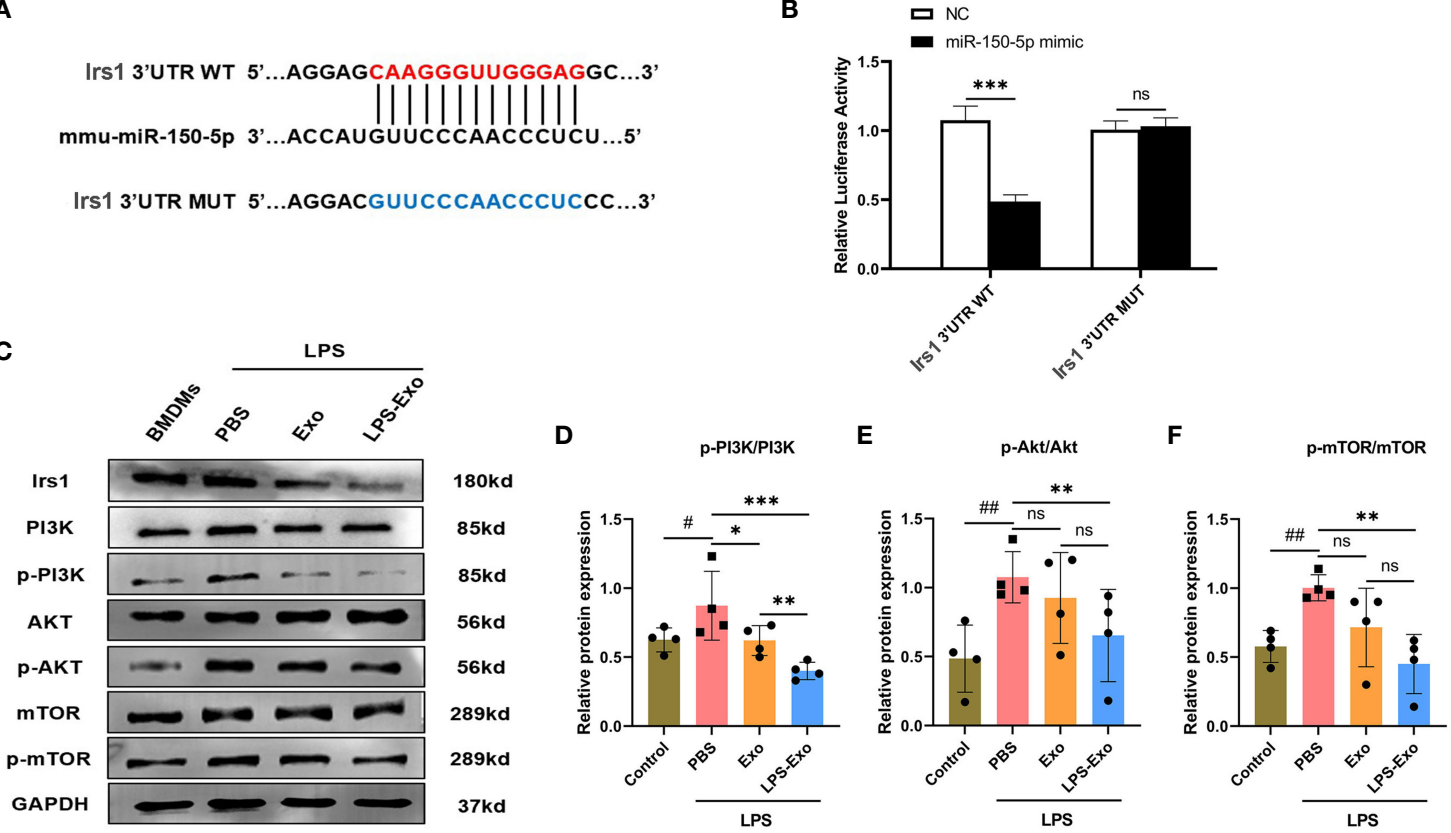
Exosomal miRNA-150–5p modulates the PI3K/Akt/mTOR signaling pathway via targeting Irs1. **(A)** The sequence alignment of miR-150–5p with its predicted target gene Irs1. **(B)** Dual-luciferase activity assay was conducted to evaluate the binding affinity between miR-150–5p and Irs1. BMDMs were stimulated with LPS (1 μg/mL) and then cultured with LPS-Exo, Exo or PBS for 24 (h) Representative images **(C)** and relative intensity of western blot analysis for Irs1, PI3K, p-PI3K, AKT, p-AKT, mTOR and p-mTOR **(D-F)** (n=4). Data are presented as the mean ± standard deviation. ^*^ *p* < 0.05, ^**^ *p* < 0.01, ^***^ *p* < 0.001, compared to the PBS group. ^#^ *p* < 0.05, ^##^ *p* < 0.01, compared to the control group. ns: p > 0.05.

**Figure 7:**
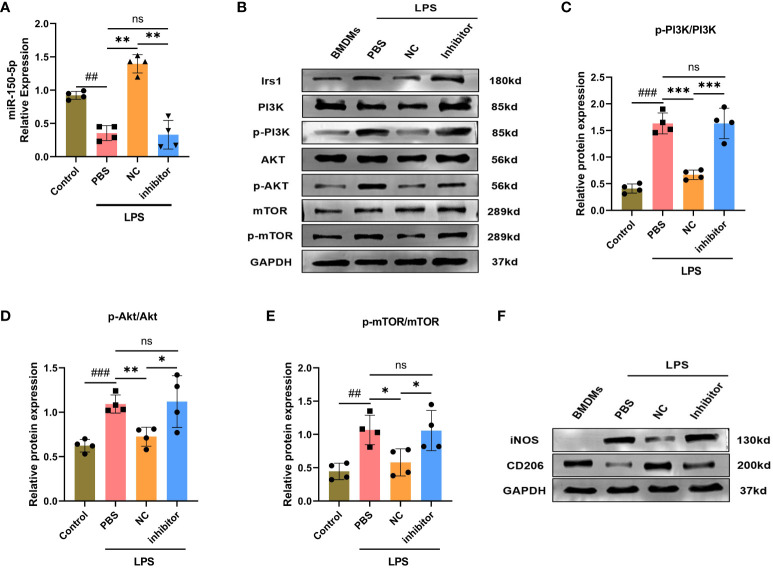
miRNA-150–5p is involved in LPS-Exo modified macrophages polarization by the PI3K/Akt/mTOR signaling pathway. BMDMs were transfected with miR-150–5p inhibitor or NC for 6 h and then cultured with LPS-Exo for 24 (h) **(A)** The transfection efficacy was verified by qRT-PCR. Representative images **(B)** and relative intensity of western blot analysis for Irs1, PI3K, p-PI3K, AKT, p-AKT, mTOR and p-mTOR **(C-E)** (n=4). **(F)** The distribution of macrophages subtype M1 (iNOS) and M2 (CD206) were measured by western blotting. Data are presented as the mean ± standard deviation. ^*^ *p* < 0.05, ^**^ *p* < 0.01, ^***^ *p* < 0.001, compared to the PBS group. ^##^ *p* < 0.01, ^###^ *p* < 0.001, compared to the control group. ns: p > 0.05.

To elucidate the mechanism through which miR-150–5p mediates macrophage polarization, bioinformatics analysis was performed to investigate the putative target genes of miR-150–5p. As predicted by miRanda and RNAhybrid databases, miR-150–5p conserved the binding site in the 3’UTR of insulin receptor substance 1 (Irs1) that was a positive regulator of the PI3K/Akt/mTOR signaling pathway (**Figure 6A**). The dual-luciferase reporter assay was conducted to validate our bioinformatic predictions, and the results indicated that luciferase activity was significantly decreased by miR-150–5p overexpression in Irs1 WT 3’UTR group, but not in MUT 3’UTR group (**Figure 6B**). Furthermore, the protein expression of Irs1 was decreased in LPS-Exo-treated macrophages, which was reversed by transfection with miR-150–5p inhibitors (**Figures 6C**, **7B**). Altogether, these data suggested that miR-150–5p could inhibit Irs1 by directly binding with the 3’UTR of Irs1.

Studies have revealed the PI3K/Akt/mTOR signaling pathway is strongly associated with macrophage polarization, which can be positive regulated by Irs1. We hypothesized that miR-150–5p modulates the PI3K/Akt/mTOR pathway by targeting Irs1, which correspondently regulating macrophage polarization. To confirm our hypothesis, we employed western blotting to examine the expression levels of key proteins in the PI3K/Akt/mTOR pathway and noticed that the phosphorylation levels of PI3K, Akt, and mTOR were decreased after LPS-Exo treatment (**Figures 6C-F**). In contrast, transfection with miR-150–5p inhibitor (**Figure 7A**) prior to culturing with LPS-Exo significantly increased the phosphorylation of PI3K, Akt, and mTOR (**Figures 7B-E**). In addition, the inhibition of miR-150–5p reversed the upregulation of M2 macrophage markers and the downregulation of M1 markers induced by LPS-Exo (**Figure 7F**). We demonstrated that LPS-Exo transferred miR-150–5p to macrophage, and modulated the polarization and inflammatory response of macrophage by down-regulating PI3K/Akt/mTOR pathway via targeting Irs1.

## 4 Discussion

Sepsis is a life-threatening condition caused by dysregulation of the host systemic immune and inflammatory response to infection, and is the leading cause of morbidity and mortality of critically ill and postoperative patients in intensive care units (2, 3). Effective treatment strategy is urgently needed to improve clinical outcomes. The data collected in our study attracted substantial attention for developing exosome-based cell-free therapeutic strategies in the treatment of sepsis and offer a novel molecular target for regulating immune hyperactivation during sepsis.

MSC-based therapy is vital for physiological maintenance and organ repair after injury (8, 9, 33). However, concerns regarding the safety of using MSCs, particularly the risk of iatrogenic tumor formation and immune rejection, remain unresolved (10, 11). Recently, the therapeutic effect of MSCs has been attributed mainly to paracrine signaling, namely, the secretion of exosomes and other bioactive molecules that influence the biological functions of neighboring cells (13, 14). Exosomes have functions similar to the parental cell, such as antimicrobial and immunomodulatory properties, and possess low immunogenicity, low tumorigenicity, and high stability, making them a superior alternative therapeutic for MSCs (34, 35). Although MSC-derived exosomes have emerged as a safe and attractive mediator of immunomodulation and regenerative effects in various diseases, their therapeutic are begin and limited. MSCs have been shown to be stimulated by a variety of different biophysical and biochemical stimuli, many of which can increase the secretion and immunomodulation abilities of MSC-derived exosomes (23–25). Increasing evidence indicates that inflammatory cytokines may enhance the secretion and the therapeutic efficacy of MSC-derived exosomes (21, 36), but little is known about a sepsis environment on exosomes secretion and their effects in sepsis. In this study, we demonstrated that LPS stimulation increased the secretion of exosomes from MSCs and that exosomes produced by LPS-stimulated MSCs possessed an apparent advantage in improving the survival rate of septic mice, reducing inflammatory response, and improving organ damages. Nevertheless, the effects of the exosomes secreted from non-stimulated MSCs were rather limited. Therefore, our data highlighted that appropriate pre-conditioning of MSCs could optimize the efficacy of MSC-derived exosomes in the treatment of sepsis.

Sepsis is characterized by an overactivation of the innate immune system in response to infection, which triggers a cytokine storm and subsequent organ failure (31). As the primary effector of inflammation, macrophage provide innate immune surveillance for all tissue in the body. They are highly plastic and can be classically activated (M1) or alternatively activated (M2) based on the inflammatory cytokines and signals in the microenvironment (4, 5). Macrophage polarization regulation is particularly important for maintaining host immune homeostasis (37, 38), and dysregulation of immune responses during sepsis enhances unrestrained M1 cells but impairs M2 cell polarization, producing pro-inflammatory mediators, thus exacerbating the progression of sepsis (6, 7). Previous studies have revealed that MSC-derived exosomes play a crucial role in inhibiting M1 polarization and promoting M2 polarization to reduce inflammation (39–41). In our study, we found that after treatment with LPS-Exo, BMDMs expressed more anti-inflammatory cytokines and fewer pro-inflammatory cytokines. Meanwhile, LPS-Exo promoted the polarization of macrophage from M1 to M2 under inflammatory stimulation *in vitro*. Therefore, we believed that exosomes from LPS-preconditioned MSCs have a superior ability to modulate the activation and balance of M1/M2 macrophages, thereby improving sepsis.

As an important medium of intracellular communication, exosome can be released by all kinds of cells and transfer bioactive molecules from the parent cells to the recipient cells to exert their modulatory effects (42, 43). Given than exosomes exert their modulatory effects primarily through the delivery of miRNAs or proteins (31), we first treated LPS-Exo with RNase and proteinase K to eliminate the effects of proteins and RNAs in LPS-Exo, respectively. We found that the effects of LPS-Exo were not affected by proteinase K, whereas RNase eliminated the protective effects of LPS-Exo, indicating that miRNAs were the key molecules responsible for the sepsis-alleviating effect of LPS-Exo. Previous studies have proven that exosomes are effective vehicles for delivering miRNAs (44, 45), which are implicated in the pathogenesis of various inflammatory diseases and emerged as novel targets for intervention therapy (20, 46). In view of the better effect of LPS-Exo on the modulation of macrophage plasticity compared to that of Exo, we suggest that LPS precondition may change the miRNA profiles of exosomes, some remarkably expressed miRNAs in LPS-Exo account for their superior immuno-modulatory properties. Therefore, the miRNA expression profiles of Exo and LPS-Exo were compared through high-throughput sequencing and bioinformatics analysis. And the results displayed that, compared to Exo, LPS-Exo had 11 downregulated miRNAs and 20 upregulated miRNAs. KEGG pathway enrichment analyses suggested that the PI3K/Akt/mTOR signaling pathway was among the 20 most enriched pathways. Among the up-regulated differentially expressed miRNAs, miR-150–5p ranked second, which was previously reported to regulate macrophages polarization. Although first detected in chronic lymphocytic leukemia, recent reports have indicated that miR-150–5p is associated with the activation and differentiation of macrophages (47–49). Indeed, we validated that miR-150–5p was highly expressed in LPS-Exo versus Exo, and transfection of miR-150–5p inhibitors into BMDMs reversed the effect of LPS-Exo on the activation and polarization of macrophages. Thus, these data indicate that when transferred into recipient macrophages by exosomes, miR-150–5p plays a crucial role in modulating recipient macrophage polarization.

miRNAs are non-coding single-stranded RNAs, which can negatively regulate gene expression by inhibiting or degrading target genes at the post-transcriptional level through directly binding with the 3’UTR of target genes (50). Bioinformatics analysis showed that Irs1 was the putative target gene for miR-150–5p, that is a key protein involved in insulin signaling (51, 52). It can bind to proteins containing SH2 domains, such as PI3K and GRB2, to mediate insulin-induced signaling and activate a range of biological processes, including glucose uptake, fat synthesis, and cell growth and differentiation (51–53). The result of dual luciferase reporter assay further confirmed that miR-150–5p can directly bind to Irs1 via base complementation. The protein expression of Irs1 was decreased in the LPS-Exo-treated macrophages, whereas miR-150–5p inhibitors reversed the expression of Irs1. Furthermore, previous studies have demonstrated that Irs1 positively modulates mTOR signaling by promoting the phosphorylation of PI3K and Akt (51, 53). In addition, the mTOR signaling pathway plays a key role in regulating energy metabolism, which controls the inflammatory response and differentiation of macrophages (54, 55). Actually, decreased phosphorylation of PI3K, Akt, and mTOR were observed after LPS-Exo treatment, which were reversed after inhibition of miR-150–5p. Moreover, the sepsis model was applied to testify the functional effect of miR-150–5p in LPS-Exo *in vivo*. Our data illustrated that miR-150–5p loss-of-function markedly deteriorated proinflammatory responses and survival rates in the experimental sepsis model treated with LPS-Exo. These data indicate that exosomal miR-150–5p targets Irs1 in recipient macrophages and subsequently modulates macrophage plasticity by down-regulating the PI3K/Akt/mTOR pathway.

## 5 Conclusions

The data obtained in our study demonstrated that the exosomes secreted by LPS-stimulated MSCs improved sepsis to a greater extent than Exo. Moreover, LPS-Exo had a better ability than Exo to promote macrophage M2 polarization *in vitro*. Further analysis demonstrated that LPS-Exo functioned primarily through transferring miR-150–5p into recipient macrophages, and modulating the macrophage phenotype by down-regulating the PI3K/Akt/mTOR pathway via targeting Irs1. Our findings strongly suggest the cell-free therapeutic strategy using exosomes derived from LPS pre-conditioned MSCs for the treatment of sepsis and propose miR-150–5p as a novel molecular target for regulating immune hyperactivation during sepsis.

## Data availability statement

The original contributions presented in the study are included in the article/**Supplementary Materials**, further inquiries can be directed to the corresponding author.

## Ethics statement

The animal study was approved by Animal Care and Use Committee of the General Hospital, Tianjin Medical University. The study was conducted in accordance with the local legislation and institutional requirements.

## Author contributions

TZ: Conceptualization, Data curation, Methodology, Writing – original draft. SiL: Conceptualization, Data curation, Methodology, Writing – original draft. TZ: Methodology, Writing – review & editing. WF: Methodology, Writing – review & editing. ShL: Data curation, Methodology, Writing – review & editing. YH: Data curation, Methodology, Writing – review & editing. XW: Data curation, Methodology, Writing – review & editing. TM: Writing – review & editing.

## Supplementary material

Supplementary Figure 1 Characterization of MSCs. **(A)** P3-MSCs *in vitro* showed typical spindle fibroblast-like morphology under light microscopy. **(B)** Positive Alcian Blue staining of MSCs assessed after osteogenic differentiation induction for 3 weeks. **(C)** Positive Oil-Red-O staining of MSCs was assessed after adipogenic differentiation induction for 2 weeks. **(D)** Immunophenotype of MSCs by flow cytometry.
