# Supplementary figures and images for: Exosome-shuttled miR-150–5p from LPS-preconditioned mesenchymal stem cells down-regulate PI3K/Akt/mTOR pathway via Irs1 to enhance M2 macrophage polarization and confer protection against sepsis
Source: Front Immunol. 2024 Jun 18;15:1397722. doi: 10.3389/fimmu.2024.1397722 (PMC11217356; doi:10.3389/fimmu.2024.1397722)

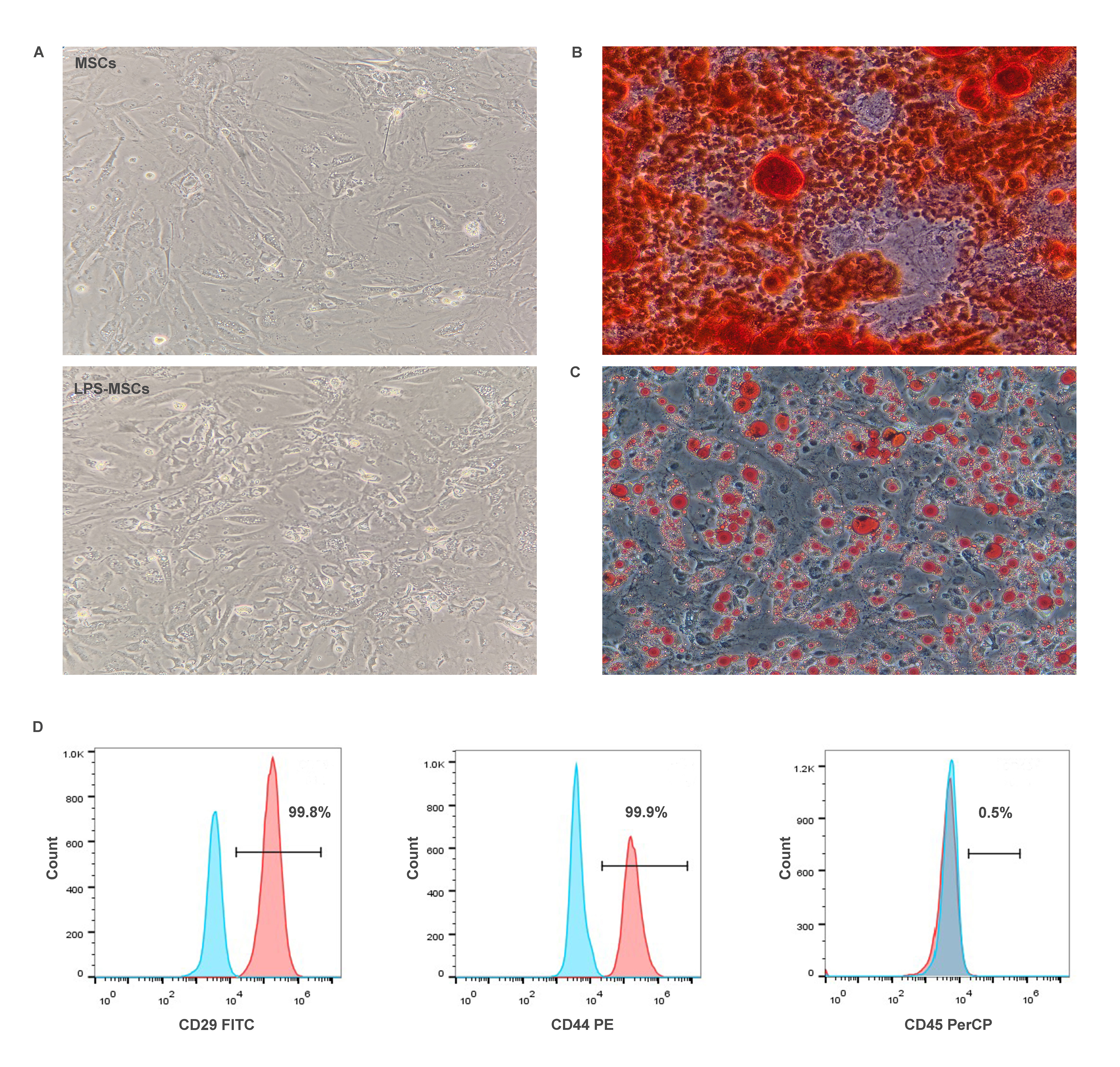

Supplement: Supplementary Figure 1 — Characterization of MSCs. (A) P3-MSCs in vitro showed typical spindle fibroblast-like morphology under light microscopy. (B) Positive Alcian Blue staining of MSCs assessed after osteogenic differentiation induction for 3 weeks. (C) Positive Oil-Red-O staining of MSCs was assessed after adipogenic differentiation induction for 2 weeks. (D) Immunophenotype of MSCs by flow cytometry. [file Image_1.jpeg]

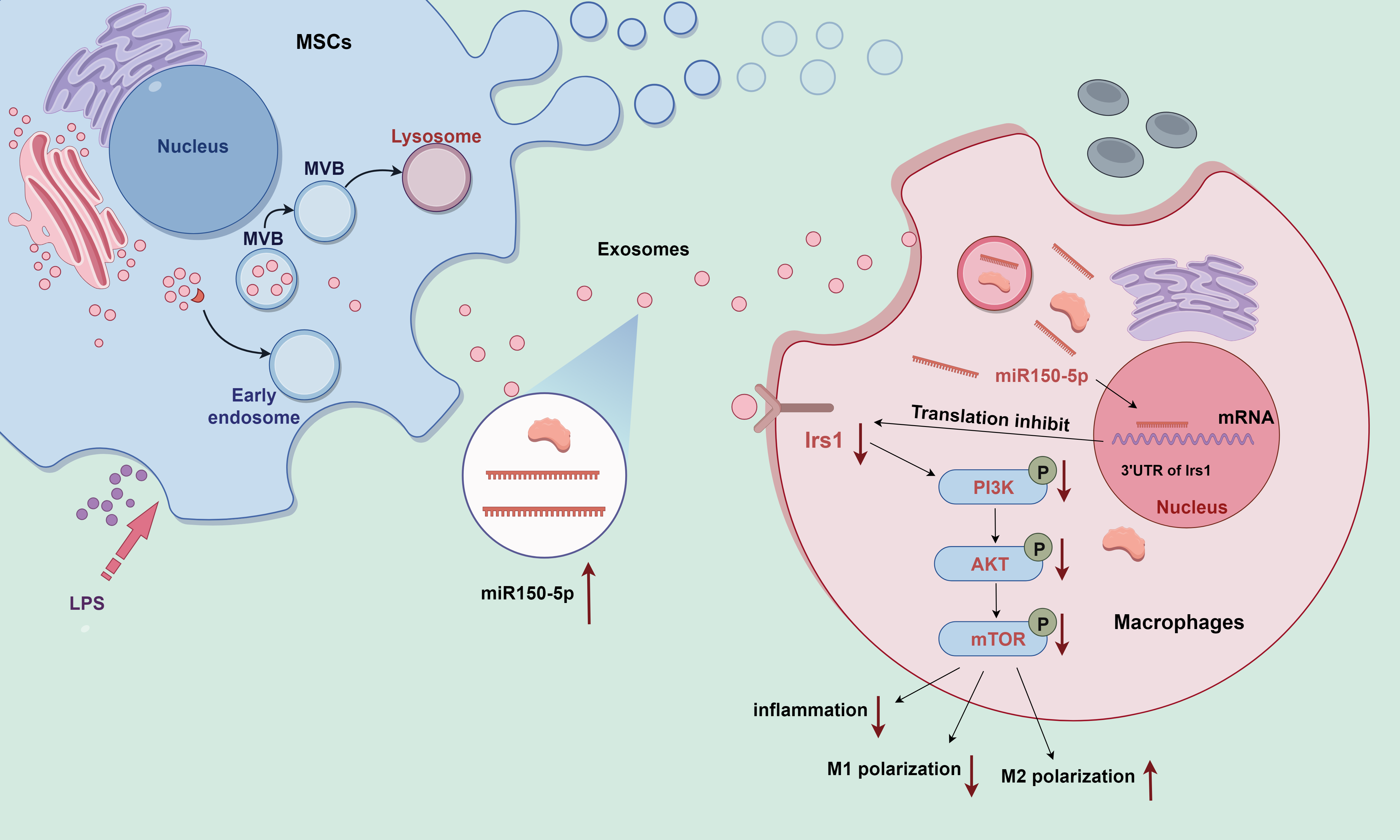

Supplement: Supplementary file 2 [file Image_2.jpeg]
